# SLiMScape 3.x: a Cytoscape 3 app for discovery of Short Linear Motifs in protein interaction networks

**DOI:** 10.12688/f1000research.6773.1

**Published:** 2015-08-05

**Authors:** Emily Olorin, Kevin T. O'Brien, Nicolas Palopoli, Åsa Pérez-Bercoff, Denis C. Shields, Richard J. Edwards

**Affiliations:** 1 School of Biotechnology and Biomolecular Sciences, University of New South Wales, Sydney, Australia; 2 UCD Conway Institute of Biomolecular and Biomedical Research, School of Medicine, University College Dublin, Dublin, Ireland; 3 Centre for Biological Sciences, University of Southampton, Southampton, UK; 4 Departamento de Ciencia y Tecnología, Universidad Nacional de Quilmes, Bernal, Argentina; 5 Fundación Instituto Leloir, Buenos Aires, Argentina

**Keywords:** SLiM, Short Linear Motif, protein-protein interaction, domain-motif interaction, minimotif, SLiMSuite, SLiMFinder

## Abstract

Short linear motifs (SLiMs) are small protein sequence patterns that mediate a large number of critical protein-protein interactions, involved in processes such as complex formation, signal transduction, localisation and stabilisation. SLiMs show rapid evolutionary dynamics and are frequently the targets of molecular mimicry by pathogens. Identifying enriched sequence patterns due to convergent evolution in non-homologous proteins has proven to be a successful strategy for computational SLiM prediction. Tools of the SLiMSuite package use this strategy, using a statistical model to identify SLiM enrichment based on the evolutionary relationships, amino acid composition and predicted disorder of the input proteins. The quality of input data is critical for successful SLiM prediction. Cytoscape provides a user-friendly, interactive environment to explore interaction networks and select proteins based on common features, such as shared interaction partners. SLiMScape embeds tools of the SLiMSuite package for *de novo* SLiM discovery (SLiMFinder and QSLiMFinder) and identifying occurrences/enrichment of known SLiMs (SLiMProb) within this interactive framework. SLiMScape makes it easier to (1) generate high quality hypothesis-driven datasets for these tools, and (2) visualise predicted SLiM occurrences within the context of the network. To generate new predictions, users can select nodes from a protein network or provide a set of Uniprot identifiers. SLiMProb also requires additional query motif input. Jobs are then run remotely on the SLiMSuite server ( http://rest.slimsuite.unsw.edu.au) for subsequent retrieval and visualisation. SLiMScape can also be used to retrieve and visualise results from jobs run directly on the server. SLiMScape and SLiMSuite are open source and freely available via GitHub under GNU licenses.

## Introduction

Many protein-protein interactions (PPIs) are mediated by a short linear motif (SLiM) in one protein interacting with globular domains in another ^1^. The past decade has seen the development of many computational methods and tools for predicting SLiMs from protein sequences and/or PPI data ^2, 3^. SLiMs are short (2–15 amino acids in length) and degenerate (with few residues determining specificity) ^4^, which makes them hard to identify against a backdrop of highly conserved structural domains. These features also impart remarkable evolutionary plasticity, and convergent (i.e. independent) evolution of new SLiM occurrences is common ^5, 6^. Some of the best examples of this come from viruses, which often hijack host cellular processes via molecular mimicry of host SLiMs ^7^. An effective approach for *de novo* SLiM discovery is to explicitly model this convergent evolution and look for enriched sequence patterns in non-homologous proteins ^5, 8, 9^. SLiMFinder combines this approach with a robust statistical model, which enables a good estimation of the probability that an observed enrichment is by chance ^6, 9– 11^. “Query” SLiMFinder (QSLiMFinder) extends this approach to define the motif search space on a specific protein, such as a viral interactor, and look for enrichment in the remaining non-homologous proteins in the dataset ^11^.

Combining motif discovery tools with biological knowledge has recently identified a new motif (“ABBA”) that binds the anaphase-promoting complex or cyclosome (APC/C) ubiquitin ligase ^12^. Nevertheless, despite the improved performance and potential of these methods, they are yet to deliver the promised windfall of new motifs ^4^. In large part, this is due to the difficulty in constructing appropriate datasets for SLiM discovery ^2, 6^. SLiM discovery relies on a careful balance of maximising the SLiM-containing signal in the data whilst removing noise by reducing the search space, either in terms of PPI or protein regions ^11^. Cytoscape is a well developed platform for the interactive generation and exploration of PPI datasets ^13^. Cytoscape is a useful resource for SLiM discovery, enabling visual groupings of proteins that share a common interaction partner and may also share a SLiM-mediated mechanism of binding. Such proteins can either be used as input for *de novo* SLiM discovery approaches ^5, 6^ or explored for enrichment of known motifs ^14^.

SLiMScape brings these tools together in a friendly environment that allows the user to browse, define and explore protein nodes whose interactors are enriched for over-represented motifs. The previously developed SLiMScape plugin for Cytoscape 2.x enabled the user to interactively run SLiMFinder ^9^ for *de novo* SLiM discovery, or SLiMSearch ^15^ for predicting novel occurrences of known SLiMs ^16^. To take advantage of the recent developments and features of Cytoscape, we have developed SLiMScape 3.x, a redesigned and updated app for Cytoscape 3.x. The SLiM discovery functions of SLiMScape have also been extended through the incorporation of QSLiMFinder ^11^ and enrichment statistics for known SLiMs using SLiMProb (formerly called SLiMSearch 1.x) ^14^. SLiMScape 3.x is built on a new set of SLiMSuite servers that permit any of the commandline options of the standalone programs to be called via the Cytoscape app. Alternatively, SLiMSuite server jobs can be executed online ( http://www.slimsuite.unsw.edu.au/servers.php) and the results imported and visualised using SLiMScape.

## Methods

### Implementation and operation

SLiMScape calls on the original Python implementations of programs in SLiMSuite; namely SLiMFinder ^9^, QSLiMFinder ^11^ and SLiMProb ^14^. These are run remotely on the SLiMSuite servers at the University of New South Wales (UNSW) through a RESTful API interface ( http://rest.slimsuite.unsw.edu.au) built on webpy 0.37 ( https://github.com/webpy). SLiMScape also includes visualisation classes that utilise the Cytoscape interface to provide a graphical representation of results.

SLiMScape was developed for Cytoscape 3, which substantially differs from previous Cytoscape versions. Because of this, it was necessary that the predecessor app, SLiMScape 1.x, be rewritten. The new version of SLiMScape was written on top of Open Service Gateway Initiative (OSGi) ^17^, a software framework of pluggable modules, using the Maven project management tool ( http://maven.apache.org) ^27^. The language used has been Java SE Runtime Environment 7 (Java 7). An active internet connection is required to submit or retrieve server jobs, although subsequent analysis can be performed offline. Cytoscape may be closed whilst jobs are queued and/or running on the server.

### Loading input data

**Figure 1.**
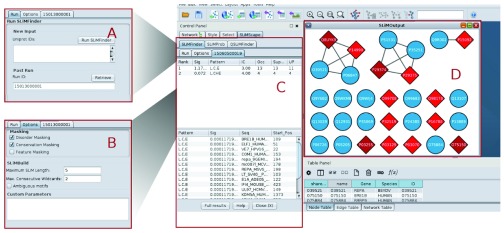
SLiMScape Cytoscape panels. **A**. SLiMFinder run panel. **B**. SLiMFinder options panel. **C**. Results panel containing sequence and motif information. **D**. Results graph. Nodes containing SLiMs are indicated as red diamonds, where dark red indicates 2+ SLiMs. Nodes without SLiMs are displayed with default settings (blue circles in this case).

SLiMScape is designed to be run directly from within Cytoscape, or to visualise the results of a previous SLiMSuite server job. As such, all three SLiMSuite programs will accept three different inputs to identify the primary dataset of proteins for analysis ( Figure 1):

1. A selection of nodes from an existing Cytoscape network view. Node ‘name’ attributes must be Uniprot identifiers or accession numbers.
2. A list of Uniprot identifiers or accession numbers, separated by commas, whitespace and/or new lines.
3. The Job ID of a previous SLiMSuite server job. This may have been submitted via SLiMScape or run directly on the server.

***SLiMFinder.*** A set of proteins is the only required input for SLiMFinder.

***QSLiMFinder.*** QSLiMFinder needs an additional query protein input, which is used to build the motif space ^11^. This should be the Uniprot accession number of one of the input proteins. If no accession number is given, the first protein returned by Uniprot will be used. This is not necessarily the same as the first accession number provided and should therefore be avoided. The query protein(s) used will be reported in the job’s log output at the server.

***SLiMProb.*** SLiMProb needs one or more SLiMs to search within the protein dataset. These should be provided as SLiM regular expressions, *e.g.* DSG.{2,3}[ST] (where .{2,3} indicates two or three “wildcards” are permitted and [ST] is a serine or threonine). (See the Eukaryotic Linear Motif (ELM) database ^18^ or SLiMScape documentation for more examples.) Multiple motifs can be provided, separated by commas. Whitespace is not permitted.

***Data aliases.*** The SLiMSuite REST servers also feature a number of input aliases ( http://rest.slimsuite.unsw.edu.au/alias). These include Uniprot ID lists and motif definitions for ELM and their occurrences from the ELM database ^18^.

### Setting parameters

The main input parameters for the SLiM programs are specified in the “Settings” panel ( Figure 1B):

- Disorder masking: masks residues with an IUPred ^19^ disorder score < 0.2. This threshold can be modified by adding an iucut=X option to the custom parameters box.
- Conservation masking: masks residues with low relative local conservation ^20^. By default, this uses GOPHER ^21^ to generate a Clustal Omega ^22^ alignment of predicted eukaryotic orthologues from the April 2015 release of the Quest For Orthologues reference proteomes ^23^. Different protein databases for GOPHER can be selected by adding an orthdb=X command to the custom parameters box. (See GOPHER server for details.)
- Feature masking: masks residues which occur in Uniprot-annotated domain and transmembrane features. A different set of Uniprot features may be masked by adding ftmask=LIST to the custom parameters box.
- SLiMBuild settings: the maximum motif length (number of defined positions and maximum wildcard spacer length) and whether to return ambiguous motifs. Amino acid equivalences for motif ambiguity can be set using the custom parameters box.
- Custom parameters: additional commandline options can be provided as “Custom parameters” ( Figure 1B). These may be used to modify/supersede the options above, or to use SLiMSuite features that have been left out of the dialogue box for clarity. Please see the SLiMSuite documentation for a full list of commandline options. New features added to the SLiMSuite servers are instantly available through the custom parameters box.

### Running the program

**Figure 2.**
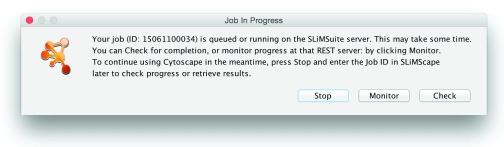
SLiMScape progress popup. Running jobs will report the server job ID for future recall and provide options to: Stop and return to Cytoscape; Monitor job progress at the SLiMSuite server; or Check for job completion.

Once all required inputs are provided and parameters are set, the desired program can be executed by clicking on the “Run X” button (where X is the name of the program being run). A popup window indicating processing will appear if a new server run is commencing ( Figure 2). Jobs typically take a few minutes to run, although larger jobs (>100 proteins) may take several hours to complete depending on server load and the program settings. The server Job ID for this run will be displayed and also populate the Job ID box in the input panel ( Figure 1A). This popup gives three progress options:

- Stop and return to Cytoscape. The job will continue running on the server but Cytoscape can be used as usual in the meantime. Additional jobs may be sent to the server whilst waiting for one to complete.
- Monitor job progress at the SLiMSuite server. This will open the job’s status page at the SLiMSuite server in the user’s default web browser.
- Check for job completion. If complete, this will load the results for visualisation. Otherwise, the popup will reload.

Alternatively, a previous server Job ID can be entered in the Job ID box and loaded by clicking the “Retrieve” button. If complete, this will load the data into Cytoscape for visualisation. Otherwise, the running popup will appear. If an inappropriate Job ID is provided, or a job has crashed, an appropriate message should appear. If in doubt, the Monitor button in the progress popup ( Figure 2) can be used to check that a job has executed correctly.

### Output

Once finished, tables showing the discovered SLiMs are presented in the Cytoscape control panel, in a new tab named after the SLiMSuite Job ID ( Figure 1C). In each case, two tables are provided: (1) a table summarising overall statistics for each motif in the dataset, and (2) details of the individual occurrences (if any) of motifs in the input proteins. Table fields are identical for SLiMFinder and QSLiMFinder, whereas SLiMProb has slightly different output ( Table 1). The Job ID produced by the server is presented in the “Job ID” box. The SLiMScape panel only shows a subset of the (Q)SLiMFinder/SLiMProb results fields; full output can be accessed at the server by clicking the “Full results” button and viewing the “main” or “occ” tabs. Results can also be accessed by entering the Job ID directly at the SLiMSuite server ( http://www.slimsuite.unsw.edu.au/servers.php), enabling results to be viewed and shared independently of Cytoscape.

**Table 1.** Main output fields for SLiMSuite summary and occurrence results tables.

Field	Description	(Q)SLiMFinder	SLiMProb
Rank	Rank of predicted SLiM	Summary	-
Sig	SLiMChance significance of predicted SLiM	Both	-
Motif	Name of SLiM being searched	-	Both
Pattern	Pattern of SLiM returned by (Q)SLiMFinder or searched by SLiMProb	Both	Summary
IC	Normalised information content of Pattern	Summary	Summary
Occ/N_Occ	No. occurrences in dataset	Summary	Summary
Support/N_Seq	No. proteins with 1+ occurrences	Summary	Summary
UP/N_UPC	No. unrelated proteins with 1+ occurrences	Summary	Summary
E_UPC	Expected number of unrelated proteins containing the motif	-	Summary
p_UPC	Probability of observing ≥ N_UPC given E_UPC	-	Summary
pUnd_UPC	Probability of observing ≤ N_UPC given E_UPC	-	Summary
Seq	Name of protein sequence	Occurrence	Occurrence
Start_Pos	Start position of occurrence	Occurrence	Occurrence
End_Pos	End position of occurrence	Occurrence	Occurrence

If a Job ID is retrieved without any nodes selected, a new network is created. By default, this will be named “SLiMOutput”; it is recommended to rename it in the network tab if multiple networks are to be analysed. Edges in this new network represent homology as detected with BLAST+ ( *E <* 1 *e* ^−4^) ^24^. The subnetworks defined by these edges correspond to the “Unrelated Protein Clusters (UPC)” used by the SLiMChance statistics to correct for evolutionary relationships and explicitly model convergent evolution.

When nodes are selected in the Cytoscape network graph, the visual representation of nodes will be updated upon results retrieval. Due to the wide range of possible applications and user requirements, SLiMScape node formatting has been kept deliberately simple to avoid confusion. SLiM presence in a node is indicated by a change in colour and shape; from the native settings to a red diamond ( Figure 1C). A darker shade of red indicates multiple SLiMs being present in that node. Only selected nodes will be altered and any nodes without SLiM occurrences remain as they were; if a network has already been formatted ( *e.g.* by an earlier SLiMScape run), the old formatting is not removed first. To remove SLiMScape formatting, select the appropriate nodes, visit the Node tab of the Style control panel and “Remove Bypass” for the relevant properties. Users can also apply additional bypass or mapping styles to manually combine results from different runs. Missing nodes will not be added to an existing network; if nodes need to be added, users should create a new network by retrieving results without any nodes selected, and then merge the networks. Clearly, this will only happen when importing results from a SLiMSuite run that was created directly on the server, or with a different network.

It should be noted that although modified node attributes will be retained, SLiMScape tabs are not saved with a Cytoscape session. It is therefore recommended to rename modified networks with the Job ID if multiple runs have been performed on the same data.

### Dependencies

The app relies on the Apache HTTP Client library to make HTTP requests to the SLiMSuite RESTful API server. The Java built in class ( java.net.HttpURLConnection) was avoided as it does not support cancellation; an element important to a responsive user interface, particularly with large data sets and the substantial processing times these require.

## Use cases

Cytoscape can be used to import PPI data from a number of databases. The IntAct database at the European Bioinformatics Institute (EBI) ^25^ is particularly suitable for SLiMScape analyses because the majority of nodes are mapped to Uniprot accession numbers. Other databases can be used but some additional mapping on to Uniprot identifiers might be required.

**Figure 3.**
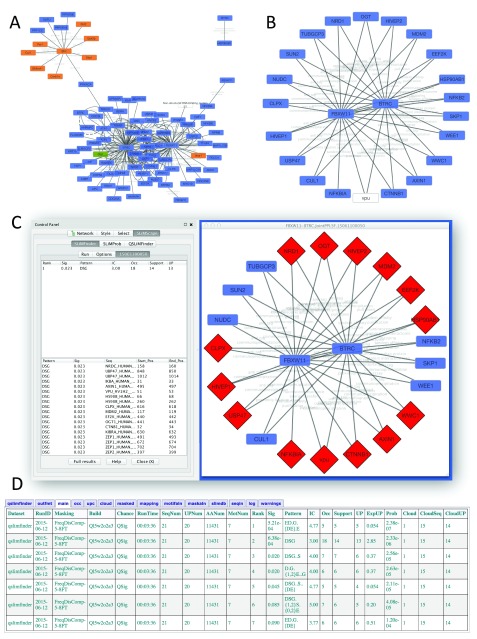
SLiMFinder results for shared FBXW11/BTRC interactors. **A**. PPI network imported from IntAct. **B**. Human and viral proteins that interact with both FBXW11 and BTRC. **C**. SLiMFinder results for *de novo* SLiM prediction in the shared interactors (Job ID 15061100050). **D**. QSLiMFinder server results for *de novo* SLiM prediction using HIV Vpu as a query (Job ID 15061200029).

The proteins ‘F-box and WD repeat domain containing 11’ (Gene Symbol: FBXW11; Uniprot: Q9UKB1) and ‘beta-transducin repeat containing E3 ubiquitin protein ligase’ (Gene Symbol: BTRC; Uniprot: Q9Y297) are two proteins of the SCF(beta-TRCP)-ubiquitin ligase complex, which recognise phosphodegron motifs (ELM DEG_SCF_TRCP1_1 ^18^) via their WD40 domains. We used the File » Import » Network » Public Databases function of Cytoscape to search for records matching FBXW11 and BTRC and imported the 184 records (184 edges; 86 nodes) from IntAct ( Figure 3A). This network was then reduced to direct human and viral PPI of BTRC and FBXW11 and duplicate edges compressed.

Working on the principle that WD40-interaction motifs are most likely to be found in proteins that interact with both WD40 proteins, Cytoscape was used to reduce the network further to nodes interacting with both proteins ( Figure 3B). These interactors were then input to SLiMFinder for *de novo* SLiM prediction, using disorder and Uniprot feature masking. SLiMFinder returned a variant of the phosphodegron motif, DSG ( *P <* 0.05), which was found in 14 of the 21 proteins ( Figure 3C).

The HIV Vpu protein interacts with both proteins and is therefore of particular interest as a potential molecular mimic. We repeated the analysis using QSLiMFinder with Vpu as the query. As previously observed ^11^, restricting the motif search space with QSLiMFinder increases the sensitivity of the search and returns the DSG motif with greater significance ( *P <* 10 ^−5^) in addition to a number of different variants of the same motif ( Figure 3D).

**Figure 4.**
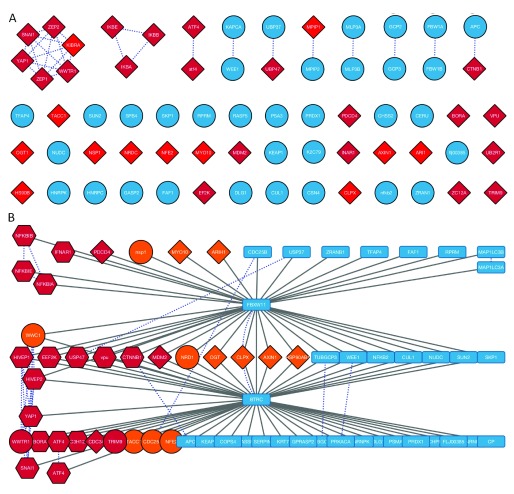
SLiMProb results for DSG and DSG.{2,3}[ST] motifs in FBXW11/BTRC interactors. **A**. Protein network generated from SLiMProb results labelled using Gene names extracted from Uniprot IDs. Edges in this network represent sequence homology. Red nodes indicate proteins with motif occurrences. Proteins with both motifs are in dark red. Proteins without either motif are blue circles. **B**. Merged PPI and homology network. Blue dotted lines represent sequence homology. Nodes are coloured and shaped according to motif occurrences: DSG, orange; DSG.{2,3}[ST], dark red; conserved DSG, circles; conserved DSG.{2,3}[ST], hexagons; no motifs, blue rectangles. The dark red circles (WWTR1 and TRIM9) have conserved DSG occurrences and unconserved DSG.{2,3}[ST] occurrences.

 These data were clearly enriched for a DSG motif, which is a more degenerate variant of the annotated ELM DEG_SCF_TRCP1_1 motif, DSG.{2,3}[ST]. To investigate this further, the full set of BTRC and/or FBXW11 interactors were subject to a SLiMProb search of both the DSG and ELM ( DSG.{2,3}[ST]) motifs with the same disorder and Uniprot feature masking (Job ID 15061200035). Using Cytoscape, proteins were arranged into three PPI sets of BTRC-only, FBXW11-only and shared interactors, arranged with DSG-containing proteins at one end and DSG-free proteins at the other ( Figure 4). The SLiMProb run was opened up as new network to identify homology between the proteins ( Figure 4A) and the two networks merged ( Figure 4B). The SLiMProb search was repeated with additional conservation masking (Job ID 15061200036). Proteins with conserved motif occurrences were manually changed to circles (conserved DSG) or hexagons (conserved DSG.{2,3}[ST]) in the merged network ( Figure 4B).

**Figure 5.**
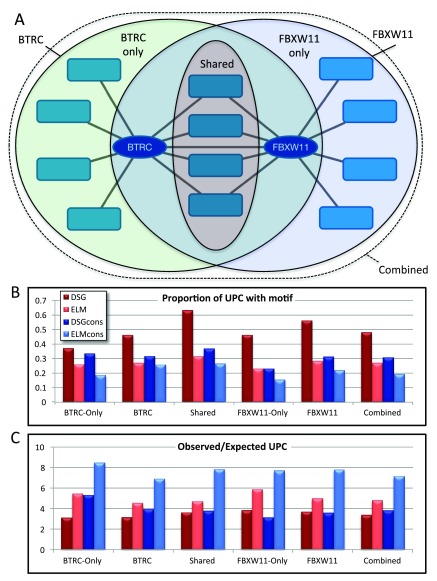
SLiMProb enrichment for DSG and DSG.{2,3}[ST] (“ELM”) motifs in FBXW11/BTRC interactors. **A**. Schematic of the different protein subsets analysed. **B**. Proportions of unrelated proteins with motif occurrences. ELM indicates DSG.2,3[ST]. DSGcons and ELMcons are conserved occurrences. **C**. Observed/expected number of unrelated proteins with motif occurrences (N_UPC/E_UPC, see Table 1).

Visual inspection of the motif distribution suggested that the DSG motif is actually more specific for the interaction than the defined ELM, showing a greater enrichment in the proportion of joint interactors versus interaction partners of only BTRC or FBXW11. This observation was confirmed by subsequent SLiMProb analysis of six different groups of proteins ( Figure 5). However, when the sequence composition of the proteins was taken into consideration, it became clear that the defined ELM was considerably more enriched across all BTRC/FBXW11 interactors than the simpler DSG motif. This was particularly pronounced when looking at evolutionarily conserved instances ( *i.e.* results from SLiMProb analyses with conservation masking) ( Figure 5C). This demonstrates the power of combining Cytoscape and SLiMSuite to get insights that are not obvious from either tool in isolation.

Also of interest is the other viral protein in the dataset, the NSP1 protein of ribovirus A (Uniprot: Q84940), which is reported to interact with FBXW11 but not BTRC. It has a DSG.SD sequence, which is intriguingly similar to the DEG_SCF_TRCP1_1 motif. Indeed, this motif was recently reported to be another case of molecular mimicry, targeting the beta-TrCP subunit ^26^. The evolutionary dynamics of SLiMs are complex; further work will need to be done to establish whether any of the other DSG instances that fail to match the more specific ELM definition represent hitherto undescribed variants of the motif or non-functional background sequence patterns.

## Summary

SLiM discovery is a challenging task that requires high quality data in addition to appropriate bioinformatics tools. There is often a disconnect between users with the biological knowledge to construct the former and those with the computational experience to run the latter. Embedding the SLiM discovery tools of SLiMSuite within the interactive environment of Cytoscape will help to bridge that gap, enable new patterns to be identified and new questions to be formulated.

## Data availability

SLiMSuite results for the figures in this manuscript can be retrieved by entering the Job ID indicated in the text through the SLiMScape app, or at: http://www.slimsuite.unsw.edu.au/servers.php. Job IDs for Figure 5 are given in Table 2.

**Table 2.** SLiMSuite Job IDs for SLiMProb analysis of DSG and DSG.2,3[ST] ( Figure 5).

Dataset	Disorder and Feature Masking	Disorder, Conservation and Feature Masking
BTRC-Only	15061200032	15061200041
BTRC	15061200031	15061200040
Shared	15061200030	15061200037
FBXW11-Only	15061200033	15061200039
FBXW11	15061200034	15061200038
Combined	15061200035	15061200036

## Software availability

### Software available from

http://apps.cytoscape.org/apps/slimscape

### Latest source code

https://github.com/slimsuite/SLiMScape

### Source code as at the time of publication

https://github.com/F1000Research/SLiMScape

### Archived source code as at the time of publication

http://dx.doi.org/10.5281/zenodo.19835 ^28^

### License

GNU Lesser General Public License ( http://www.gnu.org/licenses/gpl.html).

SLiMSuite is available via GitHub ( https://github.com/slimsuite/SLiMSuite) under a GNU General Public License (DOI: 10.5281/zenodo.19480 ^29^).

## Supplementary material

Cytoscape session file for network figures in SLiMScape manuscript. This file contains a Cytoscape 3 session file (*.cys) containing five networks used to generate Figure 3– Figure 5.
