# Supplementary material for: SLiMScape 3.x: a Cytoscape 3 app for discovery of Short Linear Motifs in protein interaction networks
Source: F1000Res. 2015 Aug 5;4:477. [Version 1] doi: 10.12688/f1000research.6773.1 (PMC4670012; doi:10.12688/f1000research.6773.1)
Supplement: Peer review history

# Peer review: Referee response for version 1

**DOI:** 10.5256/f1000research.7277.r10973

**Published:** 2015-11-09

**Authors:** Srikrishna Subramanian, Gurmeet Kaur

**Affiliations:** 1 Protein Science and Engineering, CSIR - Institute of Microbial Technology, Chandigarh, India; 2 Institute of Microbial Technology, Chandigarh, India

In their manuscript entitled “SLiMScape 3.x: a Cytoscape 3 app for discovery of Short Linear Motifs in protein interaction networks”, Olorin *et al.* have described the use of their updated SLiMScape plugin (Cytoscape 2.x) as a SLiMScape 3.x APP (Cytoscape 3.x). The effort made by the research group to actually update the plugin for use with the latest version of Cytoscape is commendable. They have also added new tools to the updated APP.

The integration of some of the SLiMSuite tools with the Cytoscape network analysis platform is likely to be of potential importance in the discovery of SLiMs. We believe that the manuscript is suitable for indexing. However, we have the following general queries:

Are there any other APPs or plugins available with Cytoscape with similar utility? If yes, then has the performance of SLiMScape been compared with those? 

On pg.4 of the version 1 of the paper, the authors refer to the “SLiMSuite documentation” for the complete list of commandline options. It would be good if the link to the documentation be added somewhere in the manuscript.

We have read this submission. We believe that we have an appropriate level of expertise to confirm that it is of an acceptable scientific standard.

# Response

**Published:** 2015-11-26

**Authors:** Richard Edwards

**Affiliations:** University of New South Wales, Australia

We are not aware of any other apps or plugins with similar utility, with the exception of the SLiMScape plugin for Cytoscape 2.x. The SLiMSuite tools made available through SLiMScape have themselves been benchmarked against other tools where possible. 

We do not currently have a single page that lists all of the SLiMSuite documentation, as each tool has its own documentation. This documentation is available via the existing links in the paper: http://www.slimsuite.unsw.edu.au/servers.php and http://rest.slimsuite.unsw.edu.au/. It can also be accessed via the SLiMSuite Blog. Finished jobs at the REST server also link directly to the relevant tool's documentation, which include commandline options.

# Peer review: Referee response for version 1

**DOI:** 10.5256/f1000research.7277.r9827

**Published:** 2015-08-14

**Authors:** Ulrich Stelzl

**Affiliations:** 1 Otto-Warburg Laboratory, Max Planck Institute for Molecular Genetics, Berlin, Germany

SLimfinder is one of the state of the art linear sequence motif discovery tool. The idea of short linear sequence motifs is intuitive, has been demonstrated as a binding mode for many domains and may govern PTMs to some extent. However, because of low sequence complexity, it is very hard to find motifs *de novo* in a reliable manner, i.e. statistically sound manner, and it is thus beyond the scope of non-motif expert biologist. Cytoscape 3 was a major change in network analysis. APPs are required that run easily and reliably and on a solid algorithmic basis, which are often computer intensive.

This manuscript reports the update of the SLIMfinder plugin to a SLiMScape APP and provides a very detailed description how to use it and a test case searching for FBXW11 and BTRC WD40 domain binding motifs “ *de novo*” (within the space of their interaction partners from INTACT). This is a very useful report for a very useful tool and the nice documentation, including the available cys sessions, will stimulate its use.

Two minor points:

1. Praxis of storage of data on the external server is a little unclear, I guess quite some potential use may find an option to make sure data are removed after analysis / after some time quite important.
2. In practice output files will be renamed by the user, but it is always nice to give unique name to files that are exported e.g. coupled to jobID or something systematic, so that many outputs can be saved e.g. as batch and then processed with a script. If all are “SLiMOutput” than this may get tedious.

I have read this submission. I believe that I have an appropriate level of expertise to confirm that it is of an acceptable scientific standard.

# Response

**Published:** 2015-08-27

**Authors:** Richard Edwards

**Affiliations:** University of New South Wales, Australia

These are excellent points and we will update the documentation accordingly. The quick/simple answers are:

1. Server data is currently stored for at least one week but longer in reality. It can be deleted sooner on request. Runs can be password protected using password=X in the “Custom parameters” box (or &password=X directly at the server). This does not encrypt the data (i.e. we can still access it directly on the server) but it will stop random access by others.

2. The SLiMSuite download includes a program, SLiMParser, that can be used to download and parse data from server runs. Basic documentation can be found here: http://rest.slimsuite.unsw.edu.au/slimparser. To parse and locally output job files, run with the commands: restin=JOBID restout=T [password=X] [restbase=X]. If restbase=X is not given, the jobid will be used for output files (JOBID.*) else they will be named X.* as set by restbase=X. Additional SLiMParser documentation will be posted on the SLiMSuite Blog.
